# Repetitive hypoxic preconditioning induces an immunosuppressed B cell phenotype during endogenous protection from stroke

**DOI:** 10.1186/1742-2094-11-22

**Published:** 2014-01-31

**Authors:** Nancy L Monson, Sterling B Ortega, Sara J Ireland, Anouk JM Meeuwissen, Ding Chen, Erik J Plautz, Erin Shubel, Xiangmei Kong, Min K Li, Laura H Freriks, Ann M Stowe

**Affiliations:** 1 Department of Neurology and Neurotherapeutics, University of Texas Southwestern Medical Center at Dallas, 6000 Harry Hines Blvd, Dallas, TX 75390-8813, USA

**Keywords:** Hypoxic preconditioning, B cells, CXCL13, Stroke, Neuroprotection, B10

## Abstract

**Background:**

Repetitive hypoxic preconditioning (RHP) creates an anti-inflammatory phenotype that protects from stroke-induced injury for months after a 2-week treatment. The mechanisms underlying long-term tolerance are unknown, though one exposure to hypoxia significantly increased peripheral B cell representation. For this study, we sought to determine if RHP specifically recruited B cells into the protected ischemic hemisphere, and whether RHP could phenotypically alter B cells prior to stroke onset.

**Methods:**

Adult, male SW/ND4 mice received RHP (nine exposures over 2 weeks; 8 to 11 % O_2_; 2 to 4 hours) or identical exposures to 21 % O_2_ as control. Two weeks following RHP, a 60-minute transient middle cerebral artery occlusion was induced. Standard techniques quantified CXCL13 mRNA and protein expression. Two days after stroke, leukocytes were isolated from brain tissue (70:30 discontinuous Percoll gradient) and profiled on a BD-FACS Aria flow cytometer. In a separate cohort without stroke, sorted splenic CD19^+^ B cells were isolated 2 weeks after RHP and analyzed on an Illumina MouseWG-6 V2 Bead Chip. Final gene pathways were determined using Ingenuity Pathway Analysis. Student’s *t*-test or one-way analysis of variance determined significance (*P* < 0.05).

**Results:**

CXCL13, a B cell-specific chemokine, was upregulated in post-stroke cortical vessels of both groups. In the ischemic hemisphere, RHP increased B cell representation by attenuating the diapedesis of monocyte, macrophage, neutrophil and T cells, to quantities indistinguishable from the uninjured, contralateral hemisphere. Pre-stroke splenic B cells isolated from RHP-treated mice had >1,900 genes differentially expressed by microarray analysis. Genes related to B-T cell interactions, including antigen presentation, B cell differentiation and antibody production, were profoundly downregulated. Maturation and activation were arrested in a cohort of B cells from pre-stroke RHP-treated mice while regulatory B cells, a subset implicated in neurovascular protection from stroke, were upregulated.

**Conclusions:**

Collectively, our data characterize an endogenous neuroprotective phenotype that utilizes adaptive immune mechanisms pre-stroke to protect the brain from injury post-stroke. Future studies to validate the role of B cells in minimizing injury and promoting central nervous system recovery, and to determine whether B cells mediate an adaptive immunity to systemic hypoxia that protects from subsequent stroke, are needed.

## Background

Repetitive hypoxic preconditioning (RHP) is a protocol that induces a naturally-protective phenotype in mice – prior to stroke onset – that defends the central nervous system (CNS) from ischemic damage months beyond the end of treatment [1]. The benefits of RHP include the attenuation of blood–brain barrier (BBB) disruption and the reduction of inflammation in the injured, ischemic hemisphere, both of which contribute to the ultimate extent of stroke damage and infarct volume [2-4]. Given that RHP is a systemic stimulus, it stands to reason that peripheral leukocyte subsets may also be altered in RHP-treated mice to minimize post-stroke inflammatory responses. In fact, we recently reported that exposing mice to a single bout of hypoxia, which induces a short-lasting period of tolerance to stroke, significantly increased peripheral B cell representations within hours in either wild-type or transgenic mouse strains [5].

The role of B cells in stroke is largely overlooked in preclinical studies, despite the fact that B cell transfer inhibits the diapedesis of T cells, neutrophils, monocytes, and macrophages into the ischemic hemisphere, concomitant with reduced infarct volumes and functional deficits, in B cell-deficient mice [6-9]. Instead, the common view remains that B cells do not contribute to the progression of stroke pathology [10-12], and therefore potential therapeutics should focus on the role of T cells in acute neurovascular injury [10,13,14]. As a result, there are also no studies investigating the post-stroke role of CXCL13, a chemokine that specifically recruits B cells during inflammation. CXCL13 is upregulated in the CNS by infiltrating immune cells at sites of multiple sclerosis lesions [15] and in preclinical studies during CNS infection [16,17]. These data, taken together with the robust B cell proliferation after one hypoxic exposure, suggest the undiscovered potential for a B cell-mediated protective immunosuppression following RHP treatment in preconditioned mice [18].

In this study, we tested the hypothesis that RHP creates a sustained, anti-inflammatory immunophenotype in protected mice. We investigated the spatial and temporal expression of CXCL13 message and protein in the post-stroke brain. We also determined the dynamics of leukocyte diapedesis into the injured CNS during ischemic tolerance to stroke using flow cytometry techniques. To establish whether RHP altered adaptive immune mechanisms in the healthy animal, we isolated splenic B cells from preconditioned and untreated mice for microarray analysis. We found an enhanced representation of B cells in the post-stroke CNS of RHP-treated mice. We also identified a novel, anti-inflammatory B cell phenotype residing for weeks after the last hypoxic exposure − an immunophenotype that may induce protection from ischemic injury in other organ systems, or suppress inflammation during other CNS pathologies. Collectively, these data reveal long-term mechanisms of endogenous CNS protection that include an immunosuppression and reprogramming of B cells occurring prior to stroke onset, with the potential for a novel, B cell-mediated protection within the ischemic cortex of RHP-treated mice.

## Methods

### Repetitive hypoxic preconditioning

The Institutional Animal Care and Use Committee at UT Southwestern Medical Center approved all procedures according to the Animal Welfare Act and PHS Animal Welfare Assurance requirements. Adult, male Swiss Webster mice (Harlan) were randomly assigned to either RHP or control groups at 6 to 7 weeks of age. During RHP, home cages were placed in hypoxic chambers and mice were exposed to stochastic hypoxic stimuli as previously described (8% or 11% O_2_; 2 or 4 hours; nine times over 2 weeks) [1], with control animals placed in identical chambers with 21% O_2_ (that is, room air) delivered for the same durations as the RHP group.

### Transient middle cerebral artery occlusion

An intraluminal suture was inserted in the common carotid artery to block blood flow to the middle cerebral artery (MCA) in anesthetized animals (2% isoflurane/70% NO_2_/30% O_2_) while body temperature was maintained at 37°C. Occlusion duration was 60 minutes. Animals were placed into an incubator (34°C) for anesthetic recovery during the occlusion period, then re-anesthetized to withdraw the occluding suture. Transcranial Laser Doppler flowmetry (Moor Instruments,Wilmington, DE, USA) was used to assess MCA occlusion at the start and end of the occlusion period, with occlusion defined as >80% reduction in MCA blood flow relative to baseline for each animal. MCA reperfusion following suture removal was defined as a return of blood flow to >50% of baseline. Mice not meeting the blood flow criteria were removed from the study at the time of surgery (17 out of 57 mice, 30%). Sham animals had common carotid artery ligation in the absence of transient middle cerebral artery occlusion (tMCAo). Mice were monitored for recovery from anesthesia, then returned to their home cages.

### Histology and confocal CXCL13 localization and quantification

Animals were sacrificed with isoflurane overdose, transcardially perfused (20 ml 0.01 M PBS, 40 ml 4% paraformaldehyde), and brains removed for cryoprotection (15% and 30% sucrose). Frozen coronal sections were prepared, blocked, and stained using standard procedures [1,5]. Primary antibodies and dilutions were: CXCL13 (initial concentration 1:20; final concentration 1:10; R&D Systems, Minneapolis, MN, USA), astrocytes (GFAP 1:200; Immunostar, Hudson, WI, USA), neurons (NeuN 1:100; Millipore, Billerica, MA, USA) and endothelial cells (CD31 1:50; BD Pharmingen, San Jose, CA, USA) [5]. Primary antibodies were detected using Alexa Fluor 488 or 594 (1:300; Invitrogen, Grand Island, NY, USA) and nuclei were counterstained with DAPI. All images were obtained using a Leica TCS SP5 confocal laser-scanning microscope (Leica, Buffalo Grove, IL, USA).

### Flow cytometry

Submandibular peripheral blood collections were pooled into heparinized blood collection tubes (3 mice/sample), resuspended in 50 μl PBS, and centrifuged at room temperature to separate serum from cells. Blood was transferred to FACS tubes, non-specific binding blocked with FcRγII/III antibody (BD Biosciences, San Jose, CA, USA) and subsequently incubated with the general leukocyte panel listed below [19] diluted in FACS buffer. Blood was then incubated in FACS Lysing Solution (1:10 in dH_2_O; BD Biosciences) at room temperature for 5 minutes, centrifuged (1,500 rpm), and washed again in FACS before analysis on the flow cytometer.

Animals were sacrificed with isoflurane overdose and transcardially perfused with 30 ml 0.01 M PBS as previously described [5] prior to brain dissection. At the time of perfusion, animals that did not clear all visible blood from the contralateral, healthy hemisphere and the sinuses were excluded from further flow cytometry analyses (4 out of 31 mice, 13 %). Tissue was processed through a 70-μm mesh screen, washed in RPMI, and lymphocytes were isolated with a 70:30 discontinuous Percoll gradient by centrifugation (2,120 g, 15 minutes, brake off). Lymphocytes were isolated from brain tissue of either the ischemic or contralateral cortex, with a pooling of three ischemic or contralateral hemispheres in each sample to ensure adequate leukocyte populations for flow analysis. The Percoll gradient does not distinguish between resident and infiltrating immune cells for either condition. After final RPMI washes, cells were resuspended in 1 ml RPMI on ice and cellularity determined by hemocytometer count for each sample. Nonspecific binding was blocked with an FcRγII/III antibody (0.5 μl/sample; BD Biosciences). The general leukocyte panel (BD Biosciences), which does not distinguish between resident activated microglia and macrophages, included CD19 (B cells; 4.0 μl/sample), CD11b (monocytes/macrophages; 0.5 μl/sample), GR1 (neutrophils; 0.5 μl/sample), CD4 and TCRβ (T cells; 0.5 μl/sample) [19]. For B cell panels, lymphocytes were isolated using Lympholyte-M (Cedarlane labs, Burlington, NC, USA), washed with FACS staining buffer, and blocked with FcRγII/III antibody. For the B-cell activation and maturation panel, cells were labeled with CD19, IgD (5.0 μl/sample), CD93 (2.5 μl/sample), CD23 (0.5 μl/sample; all eBioscience, San Diego, CA, USA) and CD45R (1.0 μl/sample), IgM (2.0 μl/sample), and CD138 (10 μl/sample; all BD Bioscience) antibodies [20-22]. For the B-regulatory panel, cells were labeled with CD5 (2.0 μl/sample; eBioscience), CD45R, CD19, CD1d (0.5 μl/sample) antibodies [9,23,24]. After a 45-minute incubation at 4°C, cells were washed with FACS wash buffer (PBS with 1% BSA, 0.22 μm filtered) and fixed in 1% paraformaldehyde (Electron Microscopy Sciences, Hatfield, PA, USA). All samples were run on a BD-FACS Aria flow cytometer using FACS Diva 6.0 software and data analyzed using FlowJo (Tree Star Inc., Ashland, OR, USA). Hemocytometer counts were used to quantify absolute number.

### Quantification of message RNA and protein expression and microarray analysis

Brain lysates were collected at time of flow cytometry analysis of cortical tissues for quantification of cortical protein expression. Quantitative immunoassays of these proteins were performed with standard ELISA kits (R&D Systems) according to the manufacturer’s instructions. Separate cohorts were transcardially perfused (20 ml RNAse-free PBS), brains removed and flash-frozen for quantitative PCR. CXCL13 mRNA expression was normalized against β-actin [1]. In a separate cohort, animals were exposed to RHP or room air and CD19^+^ B cells were sorted from spleen at 2 weeks following the last hypoxic exposure. RNA was isolated and underwent two-rounds of amplification prior to quantification on an Illumina MouseWG-6 V2 array (San Diego, CA, USA) at the UT Southwestern Microarray Core. Detectable genes were normalized (Partek Genomics Suite, St. Louis, MO, USA), with final between-group significance of the 18,099 genes detected determined via a restricted maximum likelihood analysis of variance. Gene ontology pathways were analyzed using Ingenuity Pathway Analysis.

### Carboxyfluorescein succinimidyl ester-based proliferation

As previously described, but adapted for B-cells, polyclonal responses were evaluated using the carboxyfluorescein succinimidyl ester (CFSE)-based dilution assay using bulk splenocytes from RHP or control mice [25]. Bulk cells were suspended at a 1 × 10^6^/ml concentration in PBS and incubated for 7 minutes with 0.12 μM CFSE. Cells were washed twice with serum-containing media and resuspended at 1 × 10^6^/ml concentration in culture media. Cells were activated with lipopolysaccharide (LPS) at 4 μg/ml or phorbol myristate acetate at 2 μg/ml at 37°C in 5% CO_2_ for 6 days. Subsequently, cells were washed with FACS wash buffer (PBS with 1% BSA, 0.22 μm filtered), incubated for 5 minutes at 4°C with FcRγII/III antibody (BD Bioscience) and labeled with CD19, CD45R, TCRβ and CD4 fluorescent antibodies. After 45 minutes incubation at 4°C, cells were washed with wash buffer and fixed in 1% paraformaldehyde (Electron Microscopy Sciences). Flow cytometric data were acquired using a BD-FACS Aria flow cytometer using FACS Diva 6.0 software. FlowJo 9.0 software was used to gate on Lymphocyte Gate^+^ CD19^+^CD45^+^TCRβ^-^ and polyclonal-specific responses analyzed within the CFSE-low population.

### Statistics

All studies were performed by technicians blinded to experimental condition. All between-group differences were analyzed using student *t*-test or one-way analysis of variance with Bonferroni *post-hoc* analysis (Prism). Significance was defined as *P* < 0.05.

## Results

### Repetitive hypoxic preconditioning enhances stroke-induced vascular upregulation of CXCL13 in the ischemic hemisphere

**Figure 1:**
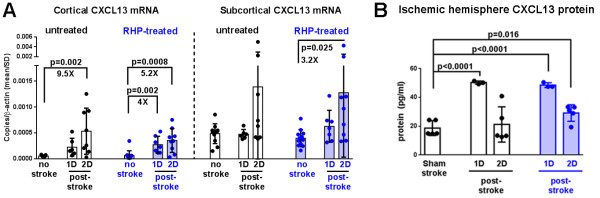
**CXCL13 is upregulated after stroke. (A)** Baseline levels of CXCL13 mRNA (n = 11), prior to transient focal stroke, were unaffected by repetitive hypoxic preconditioning (RHP; n = 12) completed 2 weeks prior in either cortical (left of dashed line) or subcortical (right of dashed line) whole brain homogenates of the same animals. RHP upregulated cortical CXCL13 message at 1 day (1D) after stroke onset (n = 6, 21 % O_2_; n = 7, RHP), though cortical values were elevated in both groups by 2 days (2D; n = 8, 21 % O_2_; n = 9, RHP). **(B)** CXCL13 protein, quantified in whole brain lysates collected during flow cytometry, shows an upregulation at 1 day following stroke (n = 3/group) that remains elevated at 2 days only in RHP-treated mice (n = 5/group) compared to sham values (n = 5/group). *P* values and fold change (#X) shown for significant values. All mRNA and ELISA experiments were run in triplicate.

The chemokine CXCL13 specifically recruits B cells for diapedesis into brain parenchyma, and is upregulated by inflammatory mechanisms in multiple sclerosis [10,15]. However, the expression of CXCL13 during CNS inflammation within the post-stroke ischemic cortex is unknown. We first sought to determine the baseline mRNA expression of CXCL13, by quantitative real-time PCR, in cortical homogenates (that is, neocortex and underlying white matter) and subcortical homogenates (for example, striatum, hippocampus, thalamus, and cerebral ventricles). In general, subcortical CXCL13 expression was higher than cortical CXCL13 expression in both untreated (that is, exposed to room air, or 21% O_2_ as designated in the Figures) and RHP-treated cohorts (both *P* < 0.0001; Figure 1A). In the absence of stroke, RHP completed 2 weeks prior did not significantly affect baseline CXCL13 expression in either cortex or subcortex compared to untreated controls. One day after stroke induction, there was a 4-fold upregulation of CXCL13 mRNA in the cortex of RHP-treated mice compared to no-stroke values (*P* < 0.05). Upregulation of CXCL13 mRNA was not significant in the cortex of untreated mice and not observed in the subcortex of either group (Figure 1A). By 2 days following stroke onset, cortical CXCL13 message was elevated in both untreated and RHP-treated groups (both *P* < 0.01).

**Figure 2:**
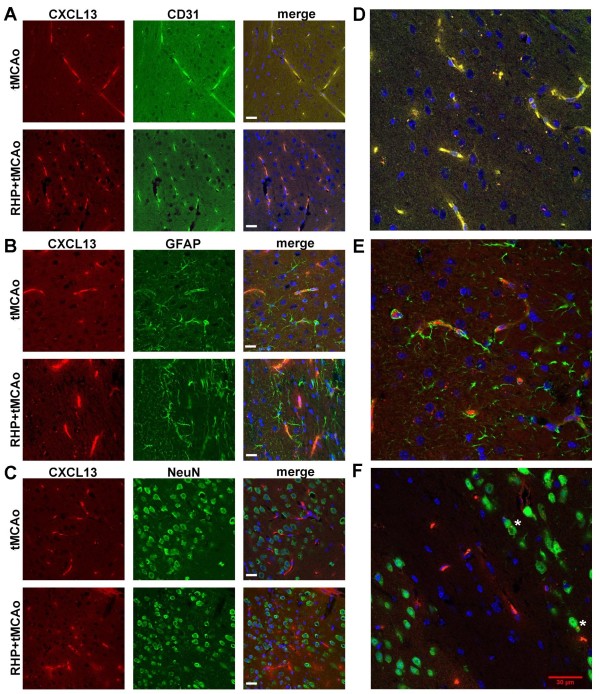
**CXCL13 is predominantly expressed in the cortical endothelium after stroke.** Representative confocal images of cortical CXCL13 protein (red; 594 nm) colocalized with **(A)** endothelial cells (CD31; green; 488 nm), **(B)** astrocytes (GFAP; green) and **(C)** neurons (NeuN; green) 24 hours following stroke at time of peak protein expression. DAPI (blue; 405 nm) was used as a nuclear counterstain and shown in the merge panel. Note the predominately vascular expression of CXCL13 at the blood–brain barrier in both control mice (top row of each pair) and repetitive hypoxic preconditioning (RHP)-treated mice (bottom row of each pair). Scale bar = 20 μm; n = 3/group; duplicate staining. Zoomed images from adjacent sections show distinct colocalization with **(D)** CD31 and **(E)** CXCL13 expression within GFAP + astrocyte endfeet. **(F)** Zoomed image of the corpus callosum (pale band within NeuN + staining) shows neurons with punctuate CXCL13 staining denoted by white asterisks. Scale bar for zoomed images = 30 μm. Images were captured using a Leica TCS SP5 confocal microscope with automated DIC optics, a 63× oil objective and LAS AF software, at 21 °C. Final figures were created using Adobe Photoshop without gamma adjustments. tMCAo, transient middle cerebral artery occlusion.

To determine if RHP affected post-stroke CXCL13 protein expression, we also analyzed cortical lysates collected from the ischemic hemispheres to measure CXCL13 protein following stroke induction. One day after stroke induction, a >1.5-fold increase in CXCL13 protein was found in the ischemic cortex, with the magnitude of expression unaffected by prior RHP (both groups *P* < 0.0001 vs sham stroke; Figure 1B). RHP-treated mice maintained a 56 % increase in cortical CXCL13 protein at 2 days following stroke (*P* < 0.05). Immunohistochemistry revealed a predominantly vascular pattern of CXCL13 protein expression throughout the ischemic brain following stroke (Figure 2). Specifically, the protein was colocalized largely to CD31^+^ endothelial cells (Figure 2A) within GFAP^+^ astrocytic endfeet at the BBB (Figure 2B,D), with minimal colocalization to astrocyte cell bodies and non-glial cells, including neurons (Figure 2C,E). Together, these data suggest an active role for the cerebral endothelium in the expression of the CXCL13 within the ischemic cortex.

### Repetitive hypoxic preconditioning maintains B cells in the ischemic hemisphere with concomitant reductions in other leukocyte subsets

**Figure 3:**
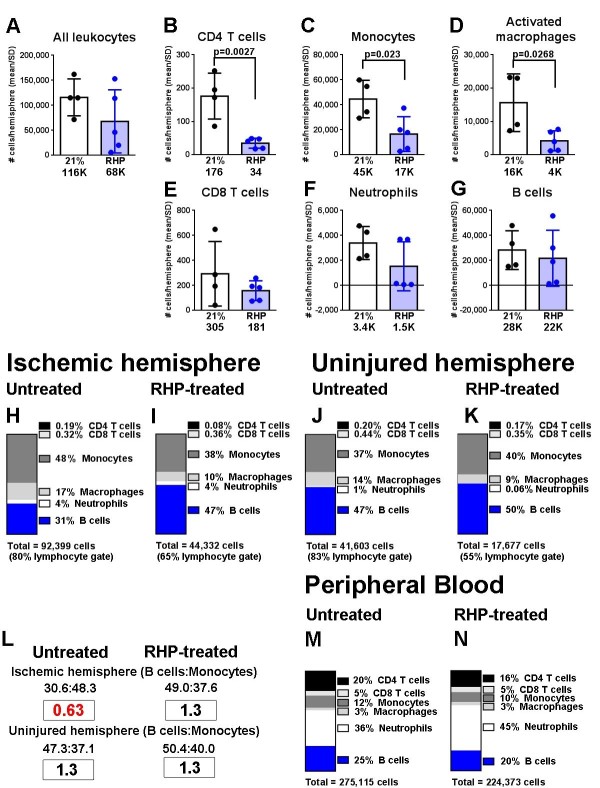
**Repetitive hypoxic preconditioning recruits B cells into the ischemic cortex following stroke. (A-G)** Overall leukocyte diapedesis was diminished in the ischemic cortex of repetitive hypoxic preconditioning (RHP)-treated mice (blue circles; n = 15) compared to control mice exposed to room air (21 % oxygen) without preconditioning (black circles; n = 12). Specifically, RHP reduced the mean populations (blue bars) of CD4+ T cells, monocytes, and macrophages in the ischemic hemispheres to levels indistinguishable from the contralateral hemispheres (shown in Additional file 2: Figure S2). Mean shown below each graph in thousands. **(H-N)** The distribution of identified leukocyte subsets from the **(H,I)** ischemic cortex and **(J,K)** contralateral (that is, uninjured) cortex (Additional file 2: Figure S2), with **(L)** ratios identified. **(M,N)** Peripheral blood of the same animals (Additional file 3: Figure S3) is shown, with total number of immune cells shown below. RHP-treated mice clearly have an increased recruitment of B cells in the ischemic cortex. Mean ± SD. Data represent four independent experiments.

To determine the impact of RHP on lymphocyte dynamics, we harvested leukocytes to investigate the post-stroke accumulation of immune cells in the injured ischemic (that is, site of tMCAo infarction) and uninjured (that is, contralateral, contralesional) hemispheres 2 days post-stroke (Figure 3; Additional file 1: Figure S1). Untreated mice had an average of 115,925 leukocytes in the ischemic brain (Figure 3A), more than double the leukocytes present in the uninjured hemisphere (50,350 leukocytes; *P* < 0.05; contralateral scatter plots and all percent distribution profiles are shown in Additional file 2: Figure S2). Of the leukocytes that diapedesed into the ischemic cortex, 92,399, or 83%, were identified by flow cytometry using our leukocyte panel, which distinguishes innate (monocytes, macrophages, neutrophils) and adaptive (T helper (CD4^+^), T cytotoxic (CD8), B cells) immune cells (Figure 3B-G). Leukocyte subsets in the untreated brain following stroke were predominantly monocytes (~48%), B cells (~31%), activated macrophages (~17%), and smaller populations of neutrophils, CD4^+^ and CD8 T cells (Figure 3H). In untreated mice, the contralateral hemisphere leukocyte subsets (Figure 3J) consisted predominantly of B cells (~47%), monocytes (~37%), and activated macrophages (~14%).

RHP treatment reduced general diapedesis into the ischemic hemisphere by 41%, to 67,918 total leukocytes (Figure 3A). Populations of CD4^+^ T cells (*P* < 0.01; Figure 3B), monocytes (*P* < 0.0.05; Figure 3C), and activated macrophages (*P* < 0.05; Figure 3D) were lowered by prior RHP to levels indistinguishable from the uninjured hemisphere (Additional file 2: Figure S2B,E,F). However, RHP minimally diminished B cell diapedesis (21,730 cells; Figure 3G), that, in conjunction with the decreased diapedesis of other subsets, increased the percent representation of B cells from 31 % in untreated post-stroke cortex to ~47% of all identified leukocytes in the protected CNS (Figure 3I). This shift toward increased B cell representation reflected the contralateral hemispheres, with only a modest RHP-induced increase from ~47% B cell representation in untreated, uninjured cortex to ~50% in RHP-treated, uninjured cortex (Figure 3K). RHP treatment therefore restored the ratio of B cells:monocytes (1.3 B cells:monocytes) [26,27] in the ischemic hemisphere compared to the untreated cohort (0.63 B cells:monocytes; Figure 3L), which was identical to the ratio in contralateral hemispheres. Minimal changes in peripheral immunophenotype were noted (Figure 3M,N; Additional file 3: Figure S3). Taken together, the analysis of percentage and counts revealed a reversal of cellularity in the ischemic cortex of RHP-treated mice similar to the pre-clinical conditions found in the uninjured cortex.

### Repetitive hypoxic preconditioning induces an immunosuppressed phenotype in resident B cells prior to stroke onset

**Figure 4:**
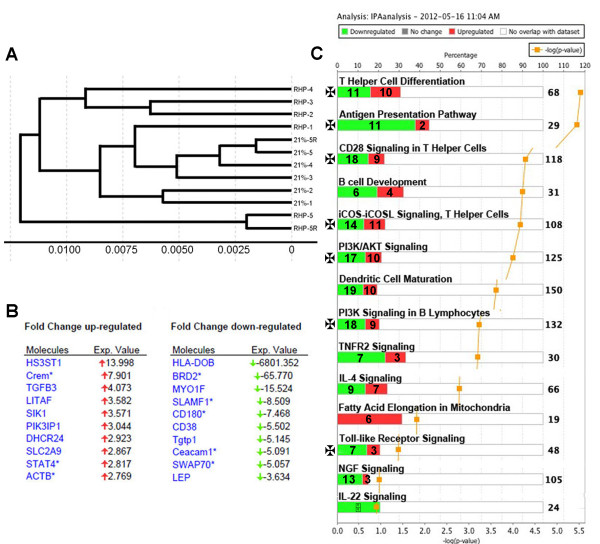
**Repetitive hypoxic preconditioning induces an immunosuppressive phenotype prior to stroke.** B cells isolated from spleen were analyzed with microarray, showing **(A)** distinct clustering of repetitive hypoxic preconditioning (RHP)-treated mice compared to controls (21 %; n = 5/group). **(B)** Top 10 up- and downregulated genes, and **(C)** top activated canonical pathways are shown. Gene products are shown in bar graph form as percentages of all genes associated with a listed pathway (upper axis). Gene numbers up/downregulated are located in the bars, with the total number of genes within the pathway shown to the right. Expression changes are shown as either upregulated (red bars) or downregulated (green bars), and pathways with B-T cell interactions are marked by a cross. Respective *P* values are indicated by orange squares (lower axis). Replicate samples on the microarray chip are indicted with (R) in panel A.

**Figure 5:**
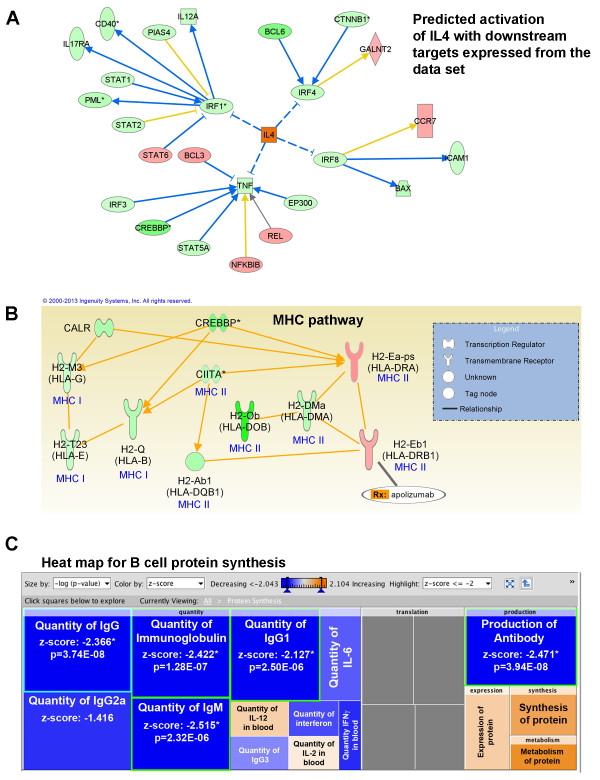
**Repetitive hypoxic preconditioning suppresses adaptive immune responses in resident B cells prior to stroke. (A)** Microarray analysis predicted a repetitive hypoxic preconditioning (RHP)-induced activation of IL-4 in B-T cell interactions to suppress downstream transcriptional regulators interferon regulatory factor (IRF)1, IRF4, and IRF8 and the cytokine TNF. Downstream molecules are coded for upregulation (red) and downregulation (green) according to expression in the data set. Blue lines lead to inhibition, whereas yellow lines indicate interactions inconsistent with the state of the downstream molecule. **(B)** Major histocompatibility complex (MHC)-related transcription and receptor responses in RHP-treated B cells. All molecules shown are significantly downregulated (green) or upregulated (red) by RHP and are identified by gene name with the human homolog in parentheses below. **(C)** Heat map showing downregulated (blue) and upregulated (orange) biological function pathways related to protein synthesis, including antibody production, in RHP-treated B cells compared to untreated control mice. Significance is only shown for pathways with z-score > −2. All significance and graphs generated by Ingenuity Pathway Analysis software.

To assess if RHP could alter B cell immunophenotype prior to stroke onset, resident splenic B cells were isolated for microarray analysis 2 weeks following the completion of RHP (Additional file 4: Figure S4). We discovered that the CD19^+^ B cells exhibited gene expression profiles with a distinct clustering (Figure 4A) from four out of five RHP-treated mice (excluding RHP #5). B cells from RHP-treated mice had altered expression of over 1,900 genes compared to 18,099 genes identified in untreated control B cells, with the top 10 up- and downregulated genes listed in Figure 4B (top 50 up- and downregulated genes listed in Tables 1 and 2). Figure 4C shows the canonical pathways activated in RHP-treated B cells, which include a predominant downregulation of genes expressed by B cells that are important for T cell differentiation and signaling. Interestingly, seven of the top canonical pathways include key components in B-T cell interactions that would significantly alter inflammatory responses. Reanalyzing the data set to focus on B-T cell interactions highlighted significant downregulation (all z scores > −2) of the quantity of T cells (*P* = 7.4E-05), differentiation of CD4 T cells (*P* = 2.6E-04), and the immune response of cytotoxic CD8 T cells (*P* = 2.1E-03). Both CD4 and CD8 T cell subtypes have been previously implicated in the progression of neurovascular injury following stroke [4,10,14] and remain downregulated in ischemic hemisphere of RHP-treated mice after stroke (Figure 3). In addition, upstream analysis showed a predicted activation of interleukin-4 (*P* = 1.5E-02; Figure 5A) and subsequent inhibition of the downstream transcriptional regulators interferon regulatory factor (IRF)-1, IRF4, IRF8, and the cytokine TNF-α.

**Table 1:** Top 50 upregulated genes isolated from repetitive hypoxic preconditioning-treated splenic B cells compared to untreated splenic B cells

**Accession**	**Symbol**	**Fold change**	***P *** **value**	**Abbreviated definition**
NM_010474.1	Hs3st1	14.00	1.38E-09	Heparan sulfate (glucosamine) 3-O-sulfotransferase 1 (Hs3st1)
NM_013498	Crem	7.90	4.02E-02	cAMP responsive element modulator (Crem), transcript variant 3
NM_013498	Crem	7.74	1.93E-02	cAMP responsive element modulator (Crem), transcript variant 3
NM_145552	Gnl2	4.70	2.78E-02	Guanine nucleotide binding protein-like 2 (nucleolar) (Gnl2)
NM_026301.1	Rnf125	4.61	4.29E-02	Ring finger protein 125 (Rnf125)
NM_027950.1	1700012B18Rik	4.35	1.76E-08	Oxidative stress induced growth inhibitor 1 (Osgin1), nuclear gene encoding mitochondrial protein
NM_013498.1	Crem	4.21	1.57E-02	cAMP responsive element modulator (Crem), transcript variant 3
AK053379	E130012P04Rik	4.13	1.34E-02	CDNA FLJ20330 FIS, CLONE HEP10296 homolog [Homo sapiens], full insert sequence
NM_009368.1	Tgfb3	4.07	2.58E-02	Transforming growth factor, beta 3 (Tgfb3)
XM_144142.3	1810045K06Rik	3.72	5.20E-04	Kelch-like 21 (Klhl21)
NM_019980	Litaf	3.58	1.05E-02	LPS-induced TN factor (Litaf)
NM_010831.1	Snf1lk	3.57	2.21E-04	Salt inducible kinase 1 (Sik1)
XM_138460.2	LOC218060	3.23	4.61E-02	-This record was discontinued.
NM_177091.2	Fndc7	3.16	3.81E-04	Fibronectin type III domain containing 7 (Fndc7), transcript variant 1
NM_177876.2	BC026744	3.16	2.07E-07	Vacuolar protein sorting 37B (yeast) (Vps37b)
NM_145222.1	B3gnt7	3.12	8.64E-11	UDP-GlcNAc:betaGal beta-1,3-N-acetylglucosaminyltransferase 7 (B3gnt7)
NM_178149.2	1500004A08Rik	3.04	1.76E-06	Phosphoinositide-3-kinase interacting protein 1 (Pik3ip1)
NM_053272	Dhcr24	2.92	4.08E-03	24-dehydrocholesterol reductase (Dhcr24)
XM_354933.1	2410015A16Rik	2.87	2.41E-02	Lymphocyte antigen 6 complex, locus K (Ly6k)
NM_145559.1	Slc2a9	2.87	1.70E-02	Solute carrier family 2 (facilitated glucose transporter), member 9 (Slc2a9), transcript variant 4
AK037598	Stat4	2.82	4.05E-02	Signal transducer and activator of transcription 4, full insert sequence
NM_007393.1	Actb	2.77	1.08E-10	Actin, beta (Actb)
NM_134109.1	Ildr1	2.76	6.72E-03	Immunoglobulin-like domain containing receptor 1 (Ildr1)
NM_011742.1	Zfp1	2.75	5.42E-03	Zinc finger protein 1 (Zfp1), transcript variant 2
AK047415	B930059G07Rik	2.75	7.90E-03	HEMATOPOIETIC CELL PROTEIN-TYROSINE PHOSPHATASE 70Z-PEP (EC 3.1.3.48)
NM_009883.1	Cebpb	2.73	3.37E-05	CCAAT/enhancer binding protein (C/EBP), beta (Cebpb)
NM_007393.1	Actb	2.65	4.10E-10	Actin, beta (Actb)
AK038223	A130087I02Rik	2.65	1.16E-02	RIKEN full-length enriched library, clone:A130087I02 product:unclassifiable, full insert sequence
XM_148086.4	LOC224137	2.64	8.14E-03	-This record was discontinued.
AK053596	E130112N23Rik	2.63	4.30E-05	RIKEN full-length enriched library, clone:E130112N23 product:hypothetical protein, full insert sequence
NM_026301.1	Rnf125	2.63	4.68E-02	Ring finger protein 125 (Rnf125)
NM_027013.1	Scnm1	2.61	1.07E-02	Sodium channel modifier 1 (Scnm1), transcript variant 1
NM_144554.1	Trib3	2.55	7.58E-03	- tribbles homolog 3 (Drosophila), validated
AK039925	A430031F07Rik	2.54	3.29E-03	RIKEN full-length enriched library, clone:A430031F07 product:hypothetical protein, full insert sequence
XM_358387.1	Crsp2	2.53	2.36E-02	Mediator complex subunit 14 (Med14), transcript variant 2
NM_172934.1	4632417D23	2.47	4.75E-04	-"This RefSeq was permanently suppressed because currently there is insufficient support for the protein."-
AK041320	Icosl	2.47	4.97E-05	Icos ligand, full insert sequence
NM_009546.1	Trim25	2.47	2.45E-03	Tripartite motif-containing 25 (Trim25)
NM_180974.1	Foxn2	2.46	1.09E-03	Forkhead box N2 (Foxn2)
NM_172442.2	Dtx4	2.42	2.23E-02	Deltex 4 homolog (Drosophila) (Dtx4)
NM_019518.2	Grasp	2.41	1.48E-04	GRP1 (general receptor for phosphoinositides 1)-associated scaffold protein (Grasp)
AK052205	D330008I21Rik	2.39	3.03E-08	RIKEN full-length enriched library, clone:D330008I21 product:hypothetical protein, full insert sequence
NM_134109.1	Ildr1	2.38	1.33E-05	Immunoglobulin-like domain containing receptor 1 (Ildr1)
NR_027901.1	2900060B14Rik	2.37	3.51E-17	RIKEN cDNA 2900060B14 gene (2900060B14Rik), non-coding RNA
	2010204O13Rik	2.36	4.00E-02	Replaced with tetratricopeptide repeat domain 19 and validated
NM_025549.1	Arrdc4	2.35	4.64E-02	Arrestin domain containing 4 (Arrdc4), transcript variant 2
AK041549	A630020I15Rik	2.32	6.38E-04	Similar to TYROSINE-PROTEIN KINASE JAK3 (EC 2.7.1.112) (JANUS KINASE 3) (JAK-3) [Mus musculus]
NM_029083.1	Ddit4	2.32	3.87E-03	DNA-damage-inducible transcript 4 (Ddit4)
AK020047	6030405P19Rik	2.29	2.67E-11	TRUNCATED BRE ALPHA A3+ ISOFORM homolog [Homo sapiens], full insert sequence
NM_009843.2	Ctla4	2.27	4.44E-03	Cytotoxic T-lymphocyte-associated protein 4 (Ctla4)

**Table 2:** Top 50 downregulated genes isolated from repetitive hypoxic preconditioning-treated splenic B cells compared to untreated splenic B cells

**Accession**	**Symbol**	**Fold change**	***P *** **value**	**Abbreviated definition**
NM_010389	H2-Ob	−6801.35	1.57E-03	Histocompatibility 2, O region beta locus (H2-Ob)
NM_010238.1	Brd2	−65.77	1.66E-03	Bromodomain containing 2 (Brd2), transcript variant 1
XM_132218.3	2310002F18Rik	−43.53	3.68E-38	Coenzyme Q2 homolog, prenyltransferase (yeast) (Coq2), nuclear gene encoding mitochondrial protein
NM_053214.1	Myo1f	−15.52	2.26E-03	Myosin IF (Myo1f)
AK037780	Slam	−8.51	2.00E-03	Signaling lymphocyte activation molecule, full insert sequence
NM_008533	Ly78	−7.47	1.60E-03	CD180 antigen (Cd180)
XM_284165.2	A730013O20Rik	−6.59	1.23E-02	Microtubule associated tumor suppressor candidate 2 (Mtus2)
XM_194143.2	LOC269542	−5.95	7.67E-06	-This record was discontinued.
NM_026216.1	1700096C12Rik	−5.71	1.07E-03	Replaced with F-box protein 4 and validated
AK043442	Cd38	−5.50	7.29E-08	CD38 antigen, full insert sequence
AK051496	D130052N13Rik	−5.37	1.86E-02	RIKEN full-length enriched library, clone:D130052N13 product:hypothetical protein, full insert sequence
AK029383	4833419P04Rik	−5.24	4.42E-07	RIKEN full-length enriched library, clone:4833419P04 product:hypothetical protein, full insert sequence
NM_011579.2	Tgtp	−5.15	2.09E-03	T cell specific GTPase 1 (Tgtp1)
NM_011926.1	Ceacam1	−5.09	4.97E-02	Carcinoembryonic antigen-related cell adhesion molecule 1 (Ceacam1), transcript variant 3
AK045295	Swap70	−5.06	1.36E-12	SWAP complex protein, 70 kDa, full insert sequence
XM_359052.1	LOC386068	−4.88	2.49E-05	-This record was discontinued.
XM_357708.1	LOC384553	−4.81	8.72E-06	-This record was discontinued.
AK085749	Ceacam1	−4.59	3.50E-02	CEA-related cell adhesion molecule 1, full insert sequence
AK080623	A830029A02Rik	−4.27	6.89E-04	RIKEN full-length enriched library, clone:A830029A02 product:hypothetical protein, full insert sequence
XM_205896.3	LOC277506	−4.23	2.27E-03	-This record was discontinued.
AK038943	A230077I10Rik	−4.18	3.45E-02	Similar to ENDOGLYCAN, full insert sequence
NM_175128	4930430F08Rik	−4.03	1.02E-02	RIKEN cDNA 4930430 F08 gene (4930430F08Rik), protein coding
XM_150371.1	1700016D02Rik	−4.01	1.45E-02	RIKEN cDNA 1700016D02 gene , protein coding
AK040329	Tactile-pending	−3.84	3.10E-04	T cell activation, increased late expression, full insert sequence
NM_177588.1	AW413632	−3.82	1.26E-02	threonine synthase-like 1 (bacterial) (Thnsl1), transcript variant 1
NM_008493.3	Lep	−3.63	6.19E-03	Leptin (Lep)
NM_145145.1	Pomt1	−3.62	8.42E-03	protein-O-mannosyltransferase 1 (Pomt1)
AK041764	Rasgrf2	−3.56	6.48E-03	RAS protein-specific guanine nucleotide-releasing factor 2, full insert sequence
AK011989	2610305J24Rik	−3.49	1.38E-03	RIKEN full-length enriched library, clone:2610305 J24 product:unclassifiable, full insert sequence
XM_359103.1	LOC386168	−3.42	1.54E-02	-This record was discontinued
AK035999	9630025O15Rik	−3.34	2.11E-02	RIKEN full-length enriched library, clone:9630025O15 product:hypothetical protein, full insert sequence
NM_028595	Ms4a6c	−3.32	1.46E-03	Membrane-spanning 4-domains, subfamily A, member 6C (Ms4a6c), transcript variant 1
NM_010797.1	Mid1	−3.31	1.55E-05	Midline 1 (Mid1), transcript variant 1
NM_024291.3	Ky	−3.22	9.05E-03	Kyphoscoliosis peptidase (Ky)
AK080934	Ly116	−3.21	9.86E-03	Membrane-spanning 4-domains, subfamily A, member 4B, full insert sequence
XM_140160.2	LOC240168	−3.20	6.52E-03	RAS, guanyl releasing protein 3 (Rasgrp3), transcript variant 2
AK034620	9430015G10Rik	−3.17	1.64E-02	RIKEN full-length enriched library, clone:9430015G10, protein coding, validated
AK089281	Traf1	−3.13	4.00E-02	Tnf receptor-associated factor 1, full insert sequence
XM_148699.3	Crebbp	−3.08	1.25E-12	CREB binding protein (Crebbp)
BC016202	Dhx30	−3.03	2.02E-02	DEAH (Asp-Glu-Ala-His) box polypeptide 30, mRNA (cDNA clone MGC:27662 IMAGE:4527765), complete cds
AK044748	A930039F13Rik	−2.98	2.72E-04	Hypothetical RNA-binding region RNP-1 (RNA recognition motif) containing protein, full insert sequence
NM_013730.2	Slamf1	−2.96	4.44E-16	Signaling lymphocytic activation molecule family member 1 (Slamf1)
NM_009744.2	Bcl6	−2.93	8.88E-16	B cell leukemia/lymphoma 6 (Bcl6)
XM_285750.2	LOC332788	−2.91	3.46E-02	-This record was discontinued
NM_177197.2	4833405L16Rik	−2.83	3.82E-03	Isopentenyl-diphosphate delta isomerase 2 (Idi2)
NM_008739	Nsd1	−2.80	1.67E-13	Nuclear receptor-binding SET-domain protein 1 (Nsd1)
NM_001039250.1	EG638695	−2.79	2.82E-02	Predicted gene 7247
NM_011538.1	Tbx6	−2.76	3.57E-03	T-box 6 (Tbx6)
XM_358291.1	LOC385575	−2.76	3.08E-08	-This record was discontinued
XM_132034.1	Pcdh7	−2.76	3.92E-02	Protocadherin 7, protein coding, validated

The RHP-induced regulation of CD4 T cell differentiation (21/68 genes differentially affected; 52% of altered genes downregulated; *P* = 3.0E-06) included a staggering 6,800-fold downregulation of histocompatibility 2, O region beta locus (H2-Ob), the homolog to human HLA-DOB (Figure 5B). HLA-DOB is selectively expressed in B cells and required for B cell antigen presentation under the transcriptional regulation of the major histocompatibility complex (MHC) class II transactivator CIITA [28], with the mouse homolog Ciita also downregulated in RHP-treated B cells (−1.68-fold; *P* = 4.6E-06). The canonical pathway with the greatest downregulation and second-highest significance was the antigen presentation pathway (13/29 genes differentially affected; 85% of altered genes downregulated; *P* = 3.5E-06). Other B cell-specific biological functions significantly downregulated (all z-scores > −2) include hematopoiesis, development and differentiation of lymphocytes, and the production of antibody (Figure 5C). These microarray data suggest that RHP suppresses the proliferation, development, and differentiation of B cells, in addition to inhibiting B-T cell adaptive immune interactions, weeks after completion of RHP and prior to stroke onset.

**Figure 6:**
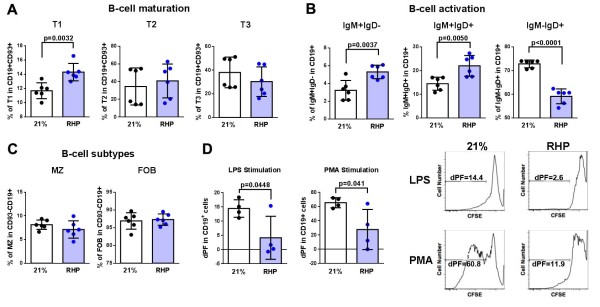
**Repetitive hypoxic preconditioning treatment modulates the peripheral B-cell immune compartment prior to central nervous system injury. (A)** Transitional state of CD19^+^CD93^+^ B-cells were quantified and measured as T1 (IgM^+^CD23^-^), T2 (IgM^+^CD23^+^) or T3 (IgM^-^CD23^+^) cells. Repetitive hypoxic preconditioning (RHP) suppresses the maturation of B-cells from T2 to T3 stage. **(B)***Ex vivo* splenic B-cell activation status was analyzed by quantifying the level of early (IgM^+^IgD^-^), mid (IgM^+^IgD^+^) or late (IgM^-^IgD^+^) CD19^+^ B-cells. RHP inhibits fully activated B-cell status in the resident B-cells. **(C)** Conventional B-cell subtypes such as marginal zone (MZ) and follicular B-cells (FOB) were quantified within the CD19^+^ CD93^-^ populations and not affected by RHP. **(A-C)** n = 6/group; two independent experiments. **(D)***In vitro* polyclonal B cell responses were assessed using carboxyfluorescein succinimidyl ester (CFSE) dilution assay with lipopolysaccharide (LPS) stimulation. Delta proliferation fraction (dPF) is the percentage of CFSE low cells in the test condition (stimulated) minus the background (non-stimulated condition). Data is representative of two independent experiments with n = 4 per condition. Mean percentages ± SD are shown. 21 %, Untreated cohorts; PMA, phorbol myristate acetate.

To gain a deeper understanding of our microarray results, we characterized splenic B cell populations from RHP-treated mice and performed an *ex vivo* phenotype analysis using flow cytometry. As B cells mature, they progressively increase their expression of MHC class II and thus increase their ability to interact with T cells [22]. We therefore evaluated the *ex vivo* maturation status of splenic B cells by first evaluating the frequency of transitional (T1, T2 and T3) B cells. T1 B cells do not migrate to lymph nodes and, while T3 B cells express higher levels of B220, they are distinct from mature B cells [22]. Gating on CD19^+^CD93^+^ B cells and using IgM versus CD23 in order to discriminate between the transitional populations (Additional file 5: Figure S5), we observed a significant increase in T1 cells isolated from RHP-treated mice compared to untreated mice (14.32% vs 11.70%, respectively; *P* < 0.01; Figure 6A). RHP therefore reduced the resident pool of B cells capable of antigen presentation, as MHC class II and CD21/CD35 expression is lowest in the T1 stage [22]. RHP also inhibits fully activated B cell status by inducing a concomitant increase in early (IgM^+^IgD^-^CD19^+^) and mid-phase (IgM^+^IgD^+^CD19^+^) B cells (3.24% vs 5.3%; *P* < 0.01, 14.52% vs 22.07%; *P* < 0.01, respectively) and decrease in late activation-phase (IgM^low^IgD^+^CD19^+^) B cells (72.88 % vs 59.17 %; *P* < 0.0001) compared to untreated mice (Figure 6B). Our microarray data also indicated a downregulation of B-T cell interactions. Evaluation of follicular B cells, which are known to require T cell help [29], showed no effect of RHP on B cell representations in the marginal or follicular zones (Figure 6C). Finally, we confirmed an RHP-induced immunosuppression of the proliferative capacity of B cells by performing an *in vitro* CFSE dilution assay. RHP-modulated B cells were incapable of responding to polyclonal stimuli such as LPS (delta proliferation fraction (dPF) = 14.48% vs 4.15%; *P* < 0.05) or phorbol myristate acetate (dPF = 65.55% vs 27.80%; *P* < 0.05; Figure 6D). Taken together, these data suggest a preferential action of RHP on B cell-mediated adaptive immune mechanisms [30] by significantly reducing mature and activated B cells in the peripheral immune compartment prior to stroke onset.

### Repetitive hypoxic preconditioning induces a regulatory B-cell population

**Figure 7:**
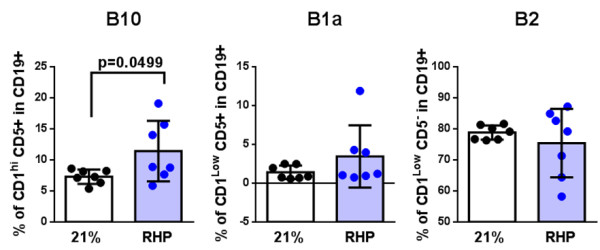
**Repetitive hypoxic preconditioning treatment specifically induces an immunosuppressive regulatory B-cell population prior to central nervous system injury.** E*x vivo* regulatory B-cell levels from repetitive hypoxic preconditioning (RHP)-treated mice relative to untreated (21 %) cohorts. B10 (CD1d^hi^CD5^+^), B1a (CD1d^+^CD5^+^) and conventional B2 (CD1d^low^CD5^-^) subpopulations were quantified within splenic CD19^+^ B-cells populations. Mean percentages ± SD are shown; n = 6/group; two independent experiments.

B10 cells (that is, regulatory B cells), with enhanced IL-10 expression, can suppress CNS disease progression for several inflammatory autoimmune diseases in both mice [6,31-33] and humans [34]. A newly emerging hypothesis [9] is that B10 cells can enhance protection from stroke-induced injury by limiting the diapedesis of other leukocyte subsets when delivered 24 hours prior to stroke onset in B cell-deficient mice [6,7]. Since this observation reflected the impact of RHP on post-stroke cortical leukocyte dynamics, we investigated whether RHP treatment induced or augmented the regulatory B cell repertoire through endogenous mechanisms prior to any CNS injury. Using the regulatory B cell gating strategy (Additional file 5: Figure S5), we observed an increase in CD1d^hi^CD5^+^ regulatory B cells in RHP-treated mice compared to untreated mice (11 % vs 7 %; *P* < 0.05; Figure 7). Conventional B1a and B2 B cell populations were unchanged. Preconditioning by repeated exposures to systemic hypoxia results in a lower disease severity [1], which correlates with a modulation of the B cell immune compartment, and suggests a novel induction of a regulatory circuitry resulting in a lower disease incidence.

## Discussion

We previously showed that RHP induced a protective phenotype from stroke-induced neurovascular injury by downregulating neuroinflammatory mechanisms within the ischemic brain [1]. In this study, we confirmed that RHP continues to attenuate neutrophil diapedesis at 2 days post-stroke and showed that the leukocyte subtypes blocked by RHP also include T cells, monocytes, and activated macrophages. In contrast, B cells are actively maintained in the ischemic hemisphere of RHP-treated mice, which correlated with an earlier upregulation of CXCL13 that, taken together with the attenuation of diapedesis, created a distribution of leukocyte subsets indistinguishable from the uninjured, contralateral hemisphere. Ratios of immune cells, and particularly B cells:monocytes, have been used to define a pathological immune microenvironment in patients with autoimmune disease [27], and more recently B cell lymphoma [26,35], though the profile for patients with stroke is currently uninvestigated. For patients with multiple sclerosis, higher disease progression was associated with higher B cell and lower monocyte numbers [27]. In contrast, our B cell:monocyte ratios within the CNS suggest that higher B cell numbers, when compared to monocyte representations, are the steady-state distribution profile within the uninjured CNS of the contralateral hemisphere that is maintained in the ischemic hemisphere of RHP-treated mice.

These findings are consistent with the emerging concept of a potential for B cell-mediated protection from stroke-induced neurovascular injury [9]. Work from Offner and colleagues show that B cell deficiency in transgenic mice increases ipsilesional leukocyte diapedesis post-stroke, while adoptive transfer [6,7] and intrastriatal injection [8] to restore B cells reduce infarct volumes and neurological deficits. These authors suggest that B cells secreting IL-10, a known post-stroke neuroprotectant [36], reduce ischemic injury by modulating subsequent neutrophil diapedesis and pro-inflammatory chemokine production [37-39]. While RHP could enhance sequestering of pro-inflammatory leukocyte subsets in other peripheral organs, including the liver [40], after stroke, we found no effect on peripheral leukocyte counts in animals with attenuated diapedesis in the ischemic hemisphere. In fact, elevated peripheral neutrophils in RHP-treated mice were actively blocked from entry into the protected CNS. Therefore, our mouse model of RHP suggests a novel intervention that creates a naturally protective phenotype that augments the potential for B cell-mediated neuroprotection [1], but without genetic or pharmacologic perturbation of the immune system.

The immunosuppressive B cell phenotype induced in RHP-treated mice (prior to stroke) suggests novel adaptive mechanisms for B cell-mediated protection from stroke. Lymphocytes encounter CNS-derived antigens upon BBB disruption and diapedesis after stroke onset, which may initiate potentially harmful, acute autoimmune responses (see reviews [18,41]). Additionally, peripheral and resident dendritic cells upregulate MHC II receptors following transient focal stroke, with peak expression occurring 3 days after stroke onset [42]. MHC II receptors colocalized with brain-derived antigen in the cervical lymph nodes and palatine tonsils of stroke patients [43], with neuronally-derived epitopes correlating with smaller infarct volumes and better functional recovery, and myelin-derived epitopes correlating with larger infarct volumes. However, immune tolerance to myelin basic protein prior to stroke improved functional recovery in mice [44]. These data highlight the complex interplay between antigen presentation and injury in the ischemic brain. Unfortunately, studies of the role of B cell antigen presentation following stroke are limited. Our microarray analysis revealed that RHP suppresses B cell antigen presentation prior to stroke in protected mice via downregulation of the master transcriptional regulator CIITA [28] and several downstream MHC I and MHC II receptors. Of note, RHP simultaneously and selectively upregulated the expression of H2-Ea-ps, a homolog for human HLA-DR (Figure 5B). Loss of HLA-DR, an MHC II receptor, in patients at 1 day after stroke is highly predictive of post-stroke infection, which impeded long-term recovery of these patients [45] and emphasizes the complexity of endogenous adaptive immunity following RHP.

Upon exiting the bone marrow, B cells are specifically recruited to the spleen where they mature to proliferate, produce antibodies and secrete cytokines when activated by antigen. When antigen is removed, a selected few remain in a sustained memory state until a rapid response is needed upon subsequent presentation of the antigen during adaptive immunity [46]. Antibody production is typically studied under states of infection or auto-immune disorders, but little is known about antibody-producing plasma cell immune responses during cerebral ischemia or endogenous neurovascular protection. In our model, the immune function with the greatest RHP-induced modulation prior to stroke was the downregulation of the antibody response. In addition, the transcriptional repressor B cell lymphoma (Bcl)-6 and Bcl-6 interacting co-repressor are both significantly downregulated (Table 2), possibly to minimize germinal center development and thus antibody-mediated immune responses [46] should injury occur. RHP decreased the repertoire of mature B cells, while increasing regulatory B cells, by increasing T1 and IgM^+^IgD^-^ B cells, with concomitant reduction of IgM^-^IgD^+^ B cells, an effect which may be occurring through either inhibition of B cell maturation and/or selective depletion of mature cells. Reduction in peripheral mature B cells (that is, IgD-expressing cells) induces tolerance in murine autoimmune studies and pan-mature B-cell depletion in human CNS diseases [47-49]. Although transitional B cells are capable of antigen presentation, they were found to be poor activators of naïve CD4 T-cells [50].

We hypothesize that RHP inhibits B cell-mediated immune mechanisms, which potentially promote acute injury, while simultaneously augmenting disease-inhibiting regulatory B cells, a complex interplay of adaptive immune mechanisms also identified in autoimmune disease [51] that will be addressed in future studies. It remains unclear whether antibodies are produced during the initial exposures to hypoxia at the start of RHP to mediate adaptive immunity resulting in subsequent post-stroke immunosuppression [18]. Furthermore, RHP is systemic stimuli that may influence B cell development in the bone marrow compartment, which serves as a contemporaneous B cell maturation and activation site to the spleen [52]. The egress of mature B cells from the bone marrow typically occurs within 5 days, a more rapid rate than during splenic maturation [52]. Therefore, our analysis of stable, immature B cell phenotypes at 14 days after RHP in the spleen most likely reflects a similar profile within the bone marrow, though this observation needs to be experimentally confirmed. Future studies will also elucidate the role of antigen presentation and antibody production in the establishment of endogenous neurovascular protection, in addition to the progression of post-stroke injury and repair.

Our findings identified the upregulation of CXCL13 within the BBB in response to stroke (Figures 1 and 2). Stroke induced a predominantly vascular expression of CXCL13 protein throughout the ischemic hemisphere, though others show both astrocytes [53] and stimulated neurons [54] can upregulate this chemokine during inflammation. While early upregulation of CXCL13 mRNA in RHP-treated mice could potentially allow for the selective recruitment of post-stroke B cells into the protected brain in the absence of other leukocyte populations, CXCL13 mRNA is equally upregulated in the untreated ischemic BBB by 2 days following stroke onset. This may prove beneficial in future studies investigating the potential for the transfer of B cell-mediated neurovascular protection to naïve mice after stroke, as selective recruitment of B cells from the periphery will not be a sole by-product of the RHP treatment. It will also improve the translation of pro-recovery B cells as a potential neurotherapeutic if we can determine if the presence of reprogrammed B cells in the ischemic cortex promotes long-term recovery mechanisms, such as neuronal plasticity and angiogenesis. Activated, but not resting, B cells are the major producers of lymphocyte-derived brain-derived neurotrophic factor in mouse and humans [54,55] and, as brain-derived neurotrophic factor secretion by B cells supports neuronal survival both *in vitro*[54] and *in vivo*[56,57], it is imperative to discover in future studies if RHP alters post-stroke B cells to enhance the upregulation of this, and other growth factors, in the post-stroke brain.

## Conclusions

With this data, we describe for the first time the expression pattern of the chemokine CXCL13 following stroke, and the novel maintenance of B cells in the protected ischemic cortex. We also identified an adaptive B cell immunophenotype following brief systemic hypoxia that includes downregulation of antigen presentation and antibody production within the quiescent immune system. In humans, living at high altitudes [58], pre-stroke exercise [59], and prior limb ischemia [60-62] all mitigate the severity of ischemic injury. It is unknown how these systemic preconditioning interventions reduce injury, though a recent clinical study shows that remote ischemia confers myocardial protection in infants through the upregulation of IL-10 [62]. Our observation of RHP-induced B cell recruitment may be the first step to understanding the role of adaptive immunity in mitigating the severity of ischemia – or other injuries – within the CNS and other organs. Ultimately, understanding the mechanisms by which the CNS chooses to protect itself is foundational to developing effective therapies, not only for the treatment of stroke-related injury, but potentially for other diseases that involve either a loss of BBB integrity or the recruitment of leukocytes into the parenchyma.

## Abbreviations

BBB: blood–brain barrier; Bcl: B cell lymphoma; BSA: bovine serum albumin; CFSE: carboxyfluorescein succinimidyl ester; CNS: central nervous system; DAPI: 4',6-diamidino-2-phenylindole; dPF: delta proliferation fraction; ELISA: enzyme-linked immunosorbent assay; Ig: immunoglobulin; IL: interleukin; IRF: interferon regulatory factor; LPS: lipopolysaccharide; MCA: middle cerebral artery; MHC: major histocompatibility complex; PBS: phosphate-buffered saline; PCR: polymerase chain reaction; RHP: repetitive hypoxic preconditioning; tMCAo: transient middle cerebral artery occlusion; TNF: tumor necrosis factor.

## Competing interests

The authors declare that they have no competing interests.

## Authors’ contributions

NLM, SJI, and AMS designed the original CNS flow cytometry experiments, which were conducted by NLM, SJI, MKL, LHF, and AMS. The B cell microarray was analyzed by AMS. B cell-specific flow cytometry panels were designed and executed by SBO, AJMM, DC, and XK. EJP and AMS conducted all tMCAo procedures, and ES conducted quantitative PCR. XK and MKL conducted protein assays and histology. SBO, SJI, DC, and AMS were responsible for data analysis. All authors read and approved the final manuscript.

## Supplementary Material

Additional file 1: Figure S1 Gating strategies for the ischemic cortex and peripheral blood samples following stroke. Gating strategies from (A) perfused ischemic and contralateral hemispheres and (B) peripheral blood. Specific leukocyte populations are identified in the gating. Click here for file

Additional file 2: Figure S2 Repetitive hypoxic preconditioning (RHP) continues to block leukocyte diapedesis into the ischemic cortex at 2 days following stroke. (A-G) Percent representation (top panel) and total count (bottom panel) shown for each gated leukocyte subset for the ischemic (filled circles) and contralateral (open circles) cortex. RHP decreased leukocyte diapedesis into the ischemic cortex of RHP-treated mice (blue symbols) at 2 days after transient middle cerebral artery occlusion (tMCAo) compared to control mice without preconditioning (21%; black symbols; n = 3 animals/point; n = 12–21 animals/group; hemocytometer counts were not collected for one experiment). RHP reduced CD4+ T-cells, monocytes, and macrophages in the ischemic hemispheres to levels indistinguishable from the contralateral hemispheres. Mean (bars) ± standard deviation (SD; whiskers); *P < 0.05; **P < 0.01; *vs contralateral cortex unless otherwise designated by a horizontal bar. Click here for file

Additional file 3: Figure S3 Repetitive hypoxic preconditioning (RHP) recruits B-cells from the periphery while actively blocking neutrophil diapedesis. (A-G) Percent representation (top panel) and total count (bottom panel) shown for each gated leukocyte subset quantified in peripheral blood. Only (F) neutrophil and (G) B-cell representations were affected by prior RHP (n = 12) compared to control (n = 24). Distribution of leukocyte subsets for cellular leukocyte counts are shown in Figure 3. Mean (bars) ± standard deviation (SD; whiskers); *P < 0.05 vs untreated mice. Click here for file

Additional file 4: Figure S4 Repetitive hypoxic preconditioning (RHP) minimally alters peripheral lymphocyte representation prior to stroke. Two weeks following RHP (blue symbols; n = 5), (A,B) leukocyte representation in peripheral blood and spleen was quantified and compared to 21% O_2_ controls (black symbols; n = 5). RHP diminished percent representation of B cells in blood, and CD8 T cells in spleen. A trend for an increase in neutrophil representation in the spleen was significant in (C) splenic leukocyte counts. While RHP-treated mice had more splenic leukocytes, (D) overall distributions of subsets were similar. B cells isolated for microarray analysis are designated by the red outline. *P < 0.05 vs 21% O_2_ controls. Data were collected on the same day for microarray analysis. Click here for file

Additional file 5: Figure S5 Flow cytometric analysis of B-cell immunophenotype after repetitive hypoxic preconditioning (RHP) modulation. (A) Gating strategies for assessment of B-cell maturation and activation in RHP-treated resident B-cells (n = 6) relative to untreated control (21%) cohorts (n = 6). (B) Gating strategy to quantify regulatory (B10 and B1a) and conventional (B2) B-cells in RHP treated mice. Data represent two independent experiments. Click here for file
