# Supplementary figures and images for: Repetitive hypoxic preconditioning induces an immunosuppressed B cell phenotype during endogenous protection from stroke
Source: J Neuroinflammation. 2014 Jan 31;11:22. doi: 10.1186/1742-2094-11-22 (PMC3926678; doi:10.1186/1742-2094-11-22)

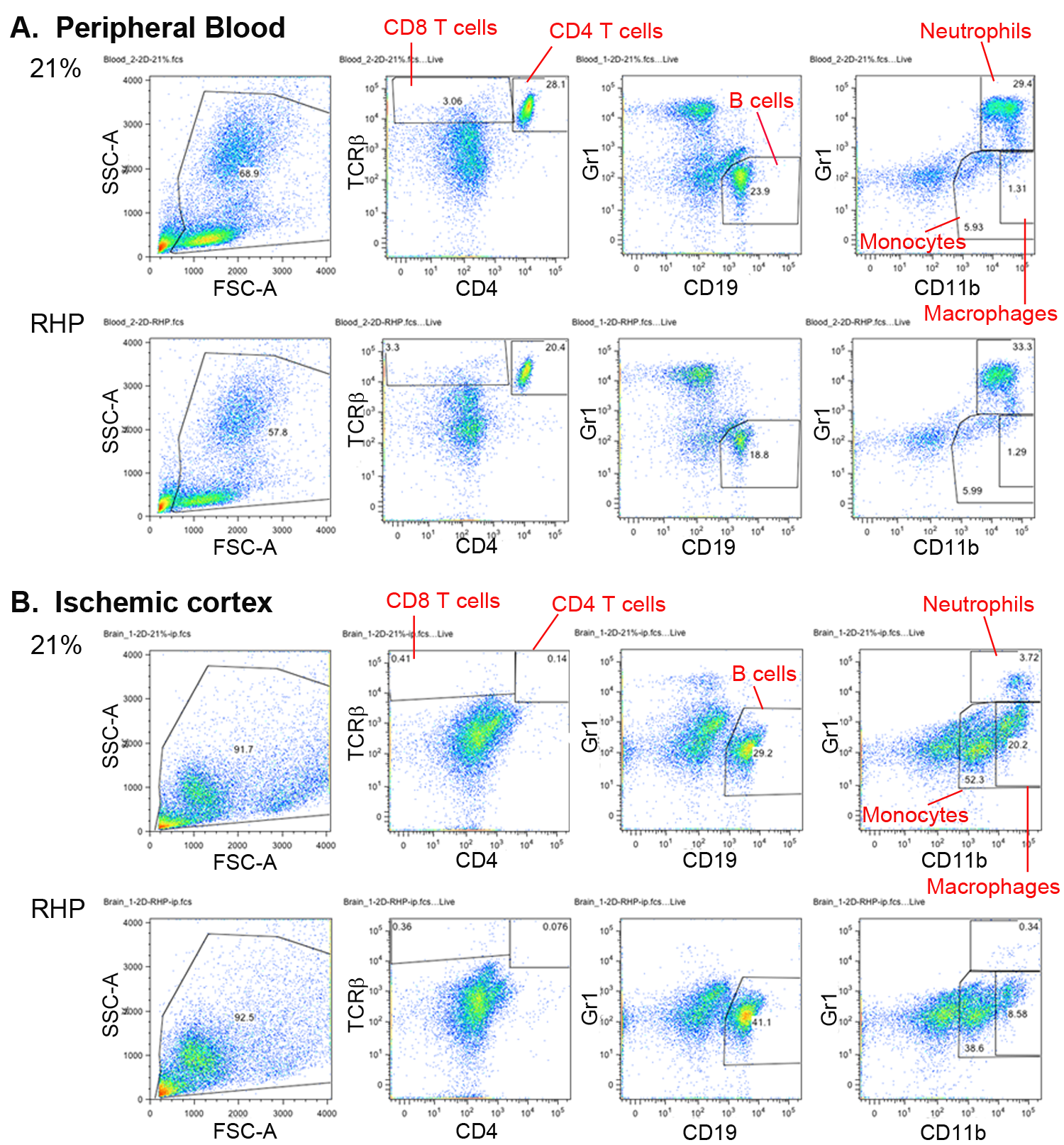

Supplement: Additional file 1: Figure S1 — Gating strategies for the ischemic cortex and peripheral blood samples following stroke. Gating strategies from (A) perfused ischemic and contralateral hemispheres and (B) peripheral blood. Specific leukocyte populations are identified in the gating. [file 1742-2094-11-22-S1.tiff]

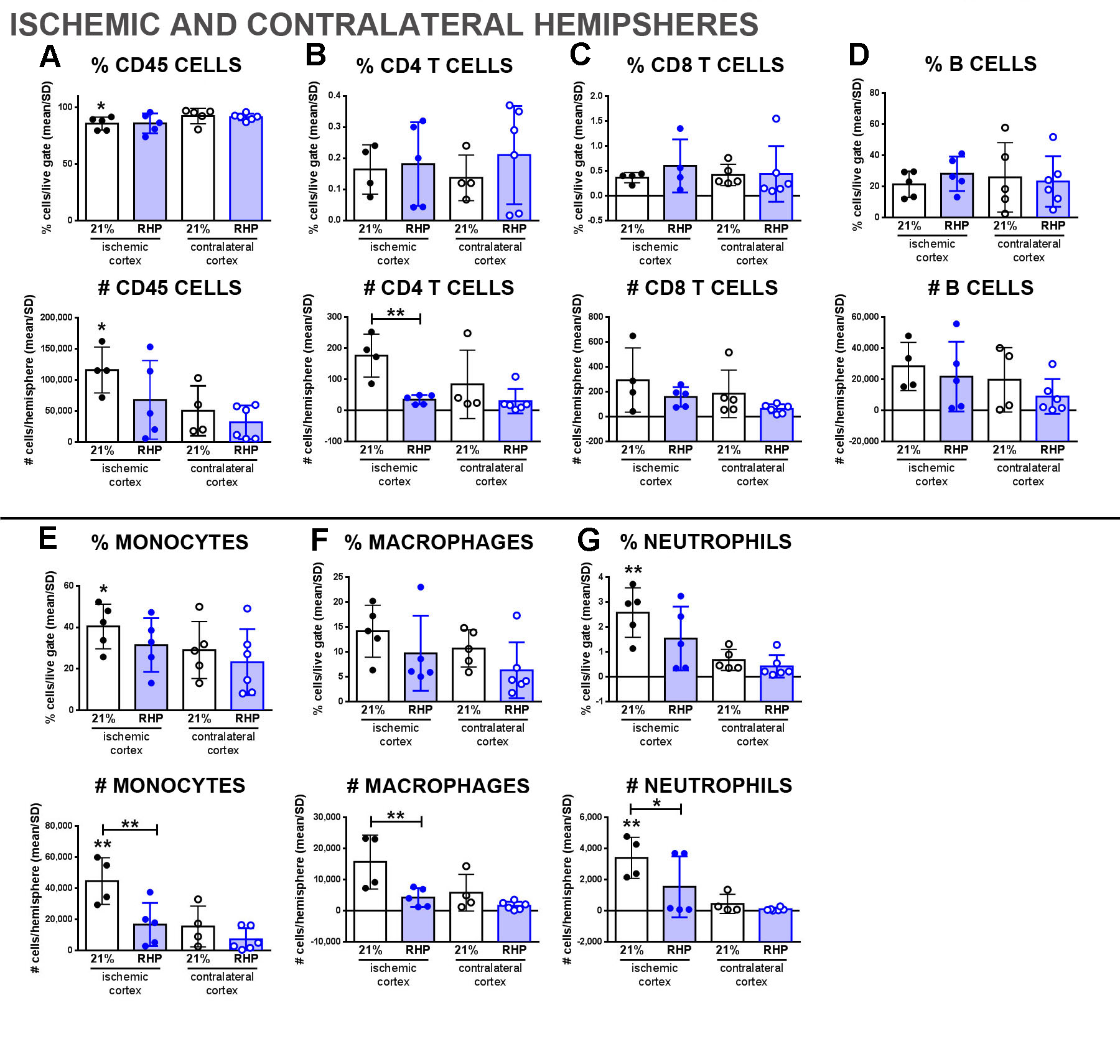

Supplement: Additional file 2: Figure S2 — Repetitive hypoxic preconditioning (RHP) continues to block leukocyte diapedesis into the ischemic cortex at 2 days following stroke. (A-G) Percent representation (top panel) and total count (bottom panel) shown for each gated leukocyte subset for the ischemic (filled circles) and contralateral (open circles) cortex. RHP decreased leukocyte diapedesis into the ischemic cortex of RHP-treated mice (blue symbols) at 2 days after transient middle cerebral artery occlusion (tMCAo) compared to control mice without preconditioning (21%; black symbols; n = 3 animals/point; n = 12–21 animals/group; hemocytometer counts were not collected for one experiment). RHP reduced CD4+ T-cells, monocytes, and macrophages in the ischemic hemispheres to levels indistinguishable from the contralateral hemispheres. Mean (bars) ± standard deviation (SD; whiskers); *P < 0.05; **P < 0.01; *vs contralateral cortex unless otherwise designated by a horizontal bar. [file 1742-2094-11-22-S2.tiff]

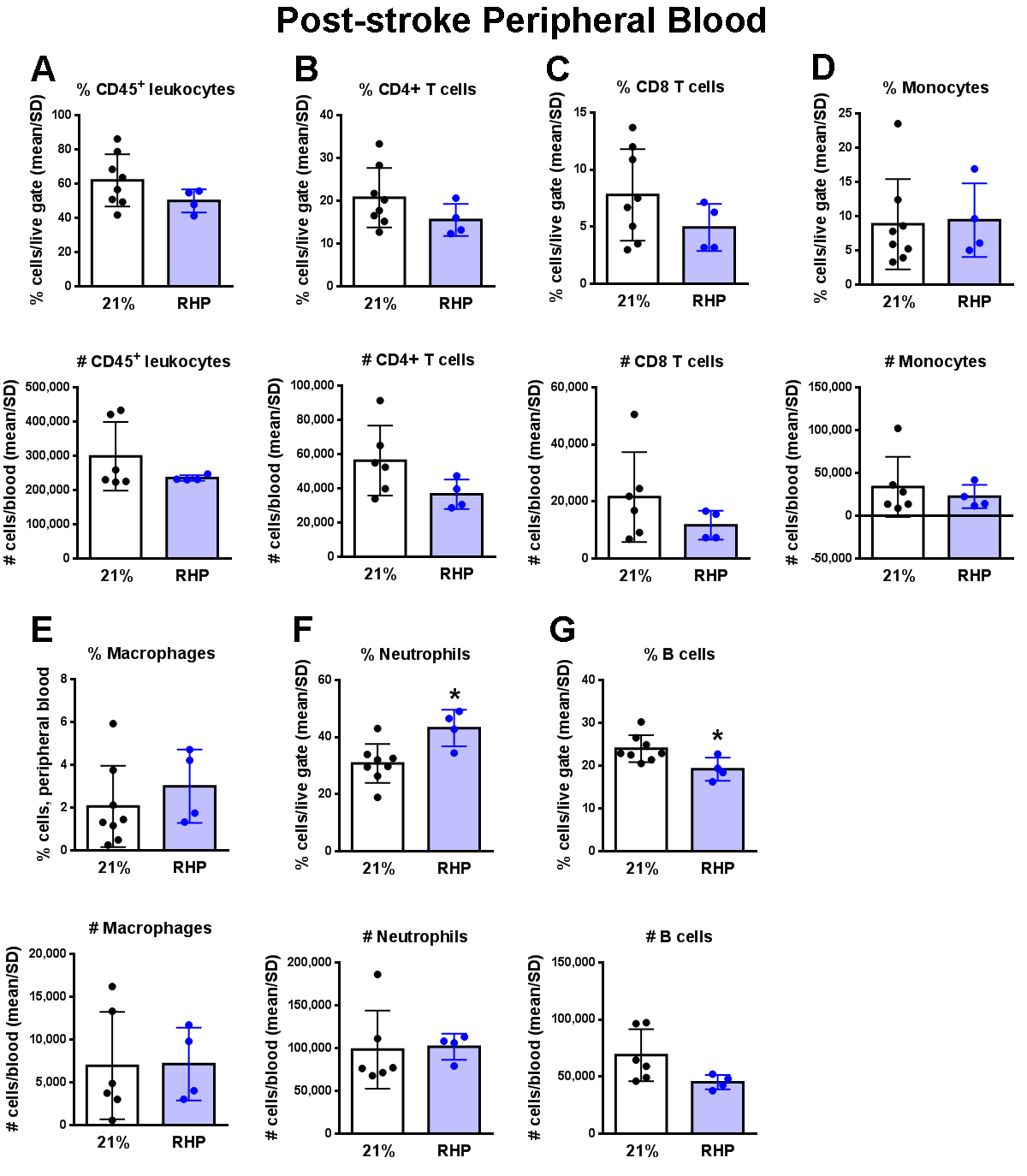

Supplement: Additional file 3: Figure S3 — Repetitive hypoxic preconditioning (RHP) recruits B-cells from the periphery while actively blocking neutrophil diapedesis. (A-G) Percent representation (top panel) and total count (bottom panel) shown for each gated leukocyte subset quantified in peripheral blood. Only (F) neutrophil and (G) B-cell representations were affected by prior RHP (n = 12) compared to control (n = 24). Distribution of leukocyte subsets for cellular leukocyte counts are shown in Figure 3. Mean (bars) ± standard deviation (SD; whiskers); *P < 0.05 vs untreated mice. [file 1742-2094-11-22-S3.tiff]

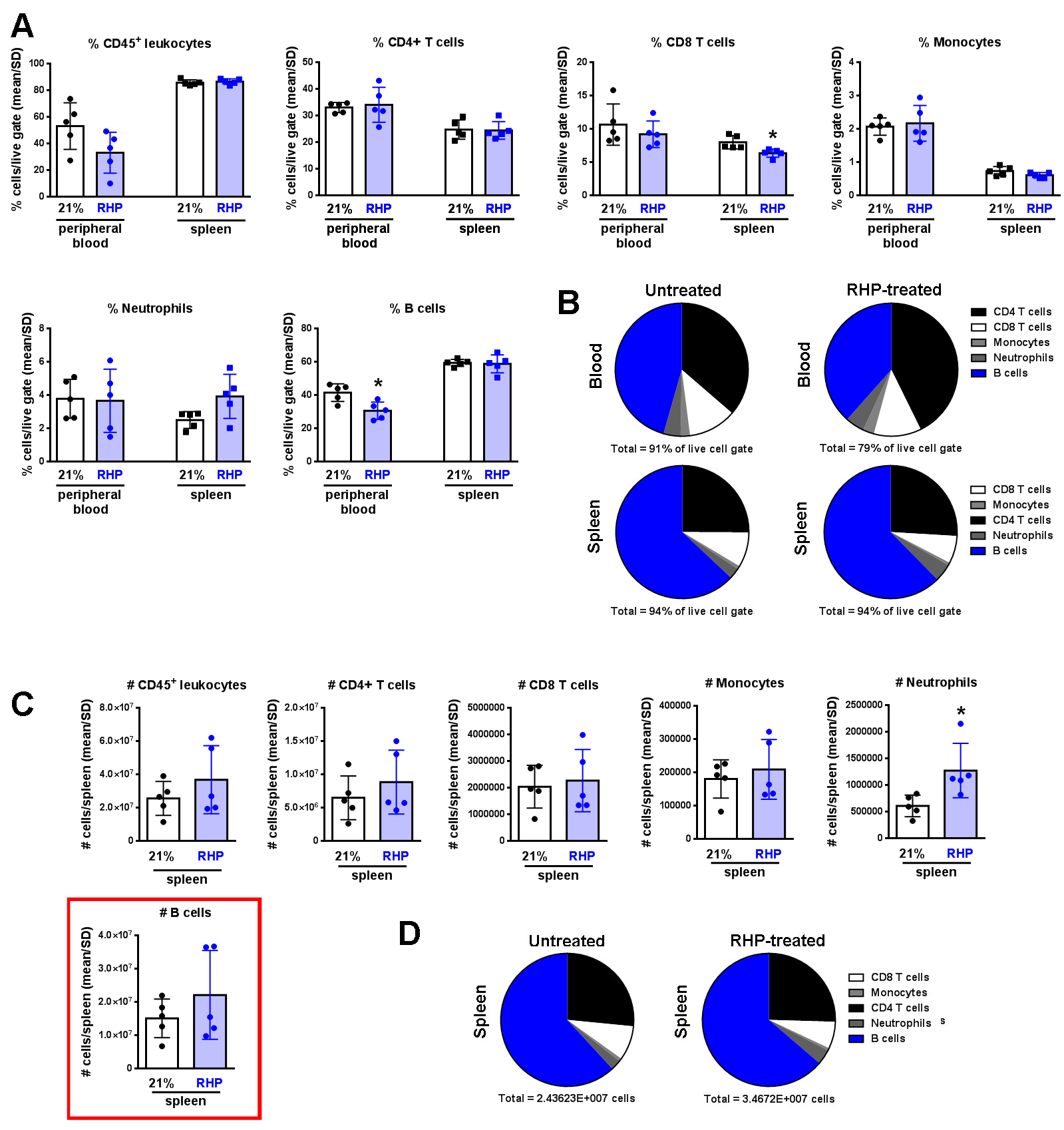

Supplement: Additional file 4: Figure S4 — Repetitive hypoxic preconditioning (RHP) minimally alters peripheral lymphocyte representation prior to stroke. Two weeks following RHP (blue symbols; n = 5), (A,B) leukocyte representation in peripheral blood and spleen was quantified and compared to 21% O2 controls (black symbols; n = 5). RHP diminished percent representation of B cells in blood, and CD8 T cells in spleen. A trend for an increase in neutrophil representation in the spleen was significant in (C) splenic leukocyte counts. While RHP-treated mice had more splenic leukocytes, (D) overall distributions of subsets were similar. B cells isolated for microarray analysis are designated by the red outline. *P < 0.05 vs 21% O2 controls. Data were collected on the same day for microarray analysis. [file 1742-2094-11-22-S4.tiff]

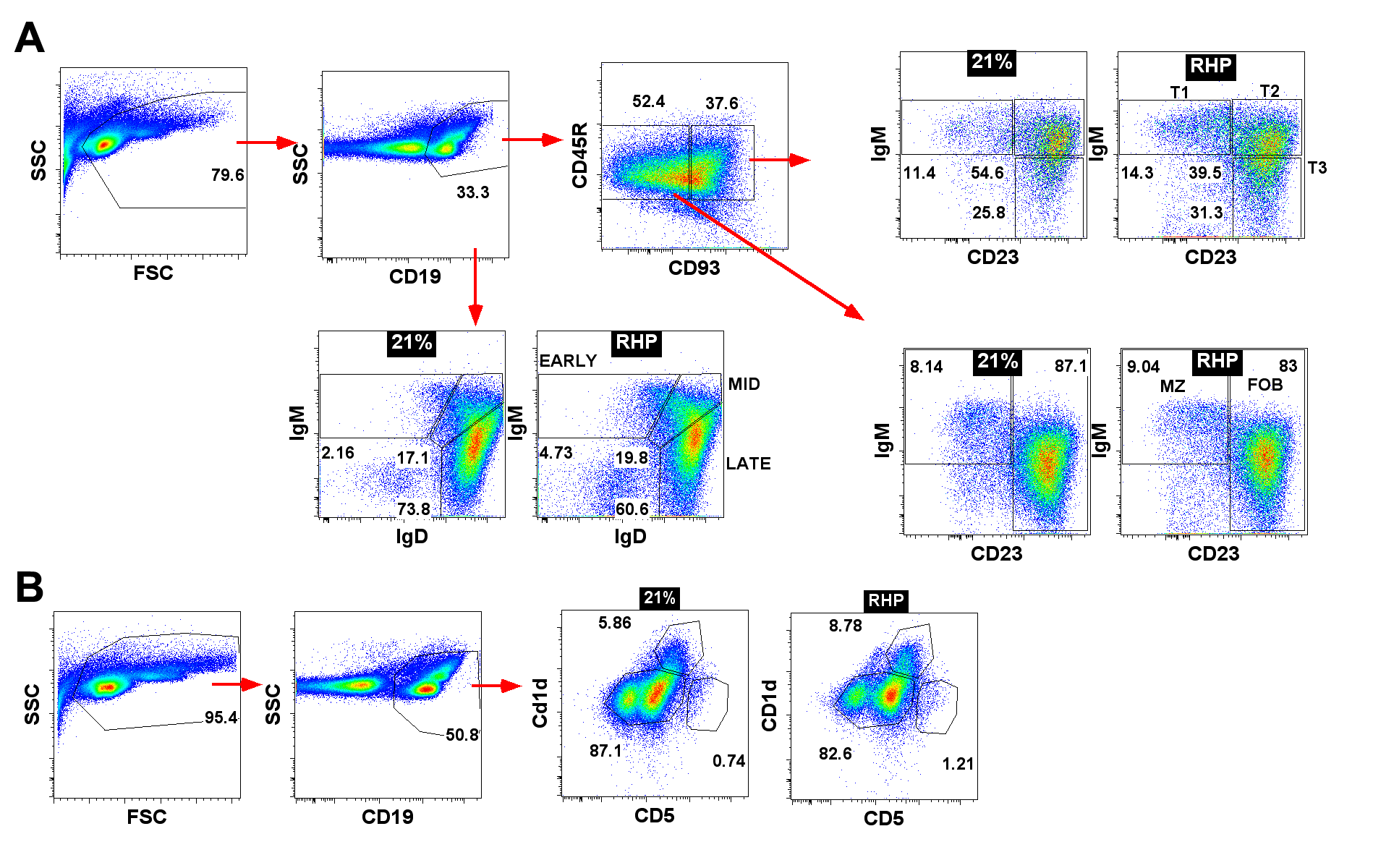

Supplement: Additional file 5: Figure S5 — Flow cytometric analysis of B-cell immunophenotype after repetitive hypoxic preconditioning (RHP) modulation. (A) Gating strategies for assessment of B-cell maturation and activation in RHP-treated resident B-cells (n = 6) relative to untreated control (21%) cohorts (n = 6). (B) Gating strategy to quantify regulatory (B10 and B1a) and conventional (B2) B-cells in RHP treated mice. Data represent two independent experiments. [file 1742-2094-11-22-S5.tiff]
